# Interleukin-6 extraction ratios during prolonged CytoSorb hemoadsorption depend on procedural blood flow

**DOI:** 10.3389/fmed.2025.1670620

**Published:** 2025-10-10

**Authors:** Jakob Gubensek, Barbara Vajdic Trampuz, Matej Zrimsek, Vanja Persic

**Affiliations:** ^1^Center for Acute and Complicated Dialysis and Vascular Access, Department of Nephrology, University Medical Center Ljubljana, Ljubljana, Slovenia; ^2^Faculty of Medicine, University of Ljubljana, Ljubljana, Slovenia

**Keywords:** interleukin 6 (IL6), cytokines, CytoSorb, hemoadsorption, reduction ratio, removal rate

## Abstract

**Background:**

Hemoadsorption with CytoSorb is a novel treatment for cytokine release syndrome, but there are few published data on the rate of cytokine removal with prolonged use. Here, we report a prospective observational study of IL-6 extraction ratios with prolonged CytoSorb use.

**Methods:**

A secondary analysis was conducted on a prospective observational cohort study involving patients treated with CytoSorb. Blood samples for IL-6 were taken before treatment, after 30 min, and every 6 h of treatment at three sites: (1) before the adsorber, (2) between the adsorber and dialyzer, and (3) after the dialyzer. The extraction ratios of the adsorber were then calculated.

**Results:**

We included 21 dialysis circuits performed in 15 critically ill patients, mainly those with cytokine storm because of septic shock. The median extraction ratio of IL-6 after 30 min was 26% (interquartile range, IQR 18–37%). The ratio decreased to 10% (6–21%) after 6 h and remained between 9–16% for up to 24 h (with a low number of circuits used beyond 12 h). Extraction ratios were similar in circuits with high (>1,000 ng/L) and low baseline IL-6 levels. On the contrary, in circuits with high blood flow (≥200 mL/min, i.e., intermittent hemodialysis), the extraction ratio was very low (median 6%) at 6 h and negligible thereafter, whereas the circuits with lower blood flow (<200 mL/min) maintained an extraction ratio of 20% for up to 12 h.

**Conclusion:**

We observed a significant reduction in the IL-6 extraction ratio within 6 h in circuits with high blood flow, whereas circuits with lower blood flow maintained an adequate extraction ratio for up to 12 h. Recent consensus recommendations on an 8–12 h exchange interval should mainly be applied to continuous dialysis methods, whereas in intermittent hemodialysis, the exchanges should be more frequent.

## Introduction

Hemoadsorption with the CytoSorb adsorber is a novel treatment option for cytokine release syndrome, which occurs primarily in sepsis but also in cardiopulmonary bypass and other conditions. The aim of hemoadsorption is to remove cytokines from the circulation and alleviate cytokine storms. Intensive research has been conducted in this field in recent years (1) with mixed data on the clinical efficacy of CytoSorb hemoadsorption. A recent human experimental endotoxemia model showed that peak interleukin-6 (IL-6) levels were reduced by half in volunteers treated with CytoSorb 15 min after the start of a 3-h lipopolysaccharide challenge (2), but most controlled clinical studies have been negative (3, 4). In addition to the clinical issues and uncertainties in optimal patient selection, there are few published data on cytokine removal, especially with the prolonged use of CytoSorb. Clinical data on IL-6 extraction ratios that would demonstrate the efficacy of the hemoadsorber are mainly available only for the first 6 h of CytoSorb treatment (2, 5, 6), whereas there are no published data on the efficacy of prolonged use of a single adsorber cartridge. In addition, as expected with any adsorption technique, cases of desorption from the adsorber back into the circulation have been described for bilirubin after 6 h of CytoSorb treatment (7) and for some cytokines (Interleukin-8 and macrophage inflammatory protein-1 alpha) within 4 h of treatment (2). A consensus statement from a group of researchers, some of whom are employed by the manufacturer of CytoSorb, recommends considering the exchange of the cartridge every 8–12 h and at the latest after 24 h of treatment (8) without providing any published evidence to support this recommendation. Similar exchange times are recommended for myoglobin removal in the case of rhabdomyolysis treatment by Hemoadsorption in the Rhabdomyolysis Task Force (9).

We report an exploratory secondary analysis of the extraction ratios of IL-6 with prolonged use of CytoSorb from a previously conducted prospective observational cohort study that was not originally designed to answer the present research question. No specific hypotheses were made regarding the IL-6 extraction ratios.

## Methods

This is a secondary analysis of a prospective observational cohort study investigating the kinetics of antibiotics in patients treated with hemoadsorption. The original study, which has not yet been published, included adult patients treated with CytoSorb (CytoSorbents Europe, Berlin, Germany) hemoadsorption for various indications between October 2022 and December 2024 at our institution who received one of the observed antibiotics. No power calculations were performed because this was a convenience sample. Written informed consent was obtained from all participants or their next of kin after enrollment in the study (delayed consent). The study was conducted in accordance with the Declaration of Helsinki and approved by the National Medical Ethics Committee (No. 0120-351/2020/8), which also approved the delayed consent because of the urgent nature of the CytoSorb treatment.

All patients were treated with either intermittent or prolonged hemodialysis (iHD) or continuous venovenous hemodiafiltration (CVVHDF) in combination with a CytoSorb adsorber, which was placed in the prefilter position. iHD was performed on a 5,008 dialysis monitor (Fresenius Medical Care, Bad Homburg, Germany) with a high-flux dialyzer from the FX series (Fresenius Medical Care, Bad Homburg, Germany) and a blood flow of approximately 250 (range 200–300) mL/min, according to the attending nephrologist’s prescription. CVVHDF was performed on a Prismaflex monitor (Gambro, Lund, Sweden) with an AN69ST series dialyzer (Gambro, Lund, Sweden) and a blood flow of approximately 150 (range 100–300) mL/min.

Regional citrate anticoagulation was administered in all cases. The study protocol included blood sampling immediately before the CytoSorb treatment, after 30 min, after 6 h, and then every 6 h of the CytoSorb treatment. Samples were collected from three sites in the circuit: (1) before the CytoSorb adsorber, (2) after the CytoSorb and before the dialyzer, and (3) after the dialyzer. IL-6 was measured immediately in the hospital laboratory in all samples, and serum was frozen for further measurement of antibiotic levels.

In this report, we present a secondary analysis of the IL-6 data. We excluded patients with unmeasurably high IL-6 levels (>500,000 ng/L) at all time points. Extraction ratios for IL-6 were calculated using the following formula: pre-CytoSorb – post-CytoSorb/pre-CytoSorb value. In a few cases where IL-6 data after CytoSorb were missing, the value after the dialyzer was used to calculate the extraction ratio. We report individual patient data and descriptive statistics. As all IL-6 data and extraction ratios at some time points were non-normally distributed, we used non-parametric statistics. Data are presented as medians with interquartile ranges (IQRs) and were compared within patients using the Wilcoxon signed-rank test and between subgroups using the Mann–Whitney *U* test. We used a simple Bonferroni correction for five comparisons (five time points); therefore, a *p*-value <0.01 (0.05/5) was considered statistically significant.

## Results

There were 24 dialysis circuits with CytoSorb adsorber included in the primary study. Three circuits were excluded as they were short-lasting, and all IL-6 levels were unmeasurable (>500,000 ng/L); 21 dialysis circuits were included in the present secondary analysis. They were performed on 15 critically ill patients with a mean age of 47 ± 18 years, 8 were male. The indication for the use of CytoSorb was sepsis in 11 patients and hemophagocytic syndrome in one patient; in these patients, the baseline IL-6 level was >500 ng/L. In 3 patients, the baseline IL-6 level was <500 ng/L, and the indications for CytoSorb use were hyperbilirubinemia, rhabdomyolysis, and leukemia with capillary leak syndrome. The median baseline IL-6 level was 4,236 (IQR 513–27,796; *N* = 15) ng/L. CytoSorb hemoadsorption was combined with iHD in 11 circuits and with CVVHDF in 10 circuits.

**Figure 1:**
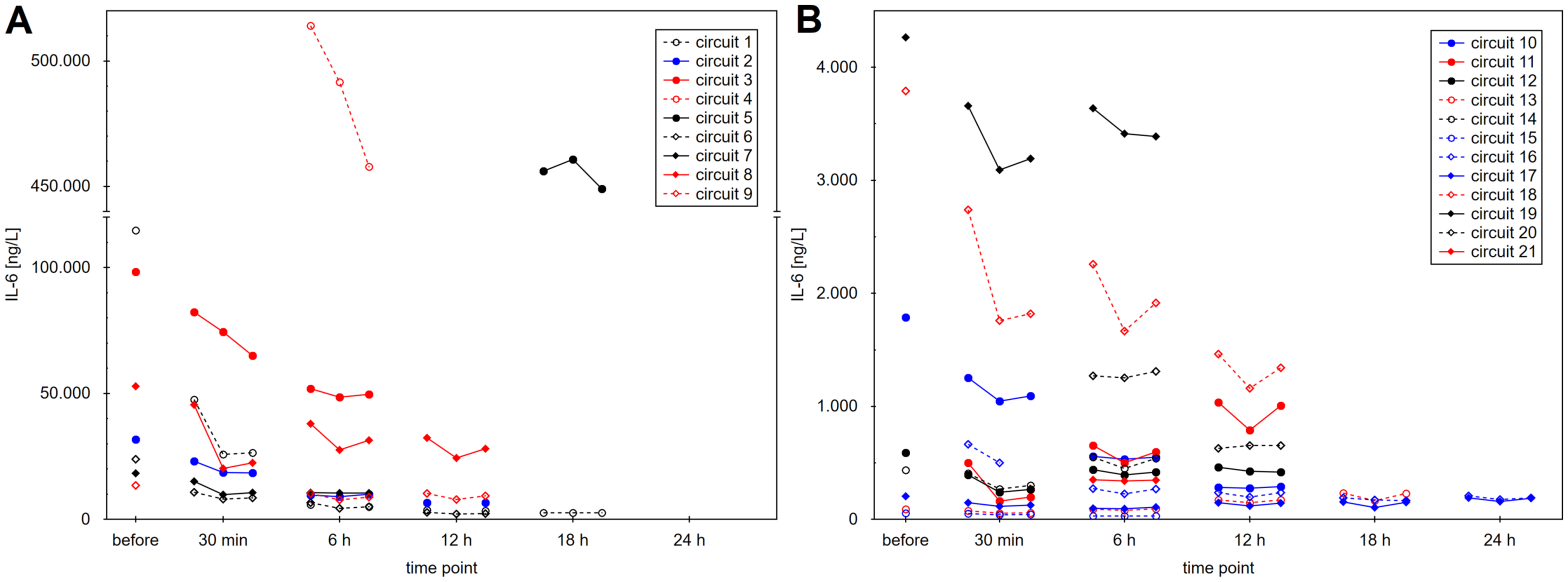
Interleukin-6 (IL-6) levels during treatment in individual dialysis circuits (patients were treated, not circuits). **(A)** Shows circuits with baseline IL-6 levels of >5,000 ng/L. **(B)** Shows circuits with lower baseline IL-6 levels for better visibility. Three values are shown at each time point: (1) before the CytoSorb adsorber, (2) after the CytoSorb but before the dialyzer, and (3) after the dialyzer.

IL-6 values of the individual circuits are shown in Figure 1 and are summarized in Table 1. IL-6 levels decreased significantly across the CytoSorb adsorber at 30 min and 6 h; the decrease at 12 h was borderline, whereas the decrease at 18 h and 24 h was not significant, but the number of circuits was small. In the vast majority of cases, we observed a slight increase in IL-6 levels across the dialyzer, most likely because of ultrafiltration and hemoconcentration (Figure 1).

**Table 1:** Summary of interleukin-6 (IL-6) levels before and after the CytoSorb adsorber and corresponding extraction ratios.

	30 min	6 h	12 h	18 h	24 h
No. of running circuits	21	21	13	5	3
No. of circuits with available data	16	19	13	5	2
IL-6 before CytoSorb (ng/L)	1,996 (399–19,061)	1,764 (393–9,973)	1,036 (283–3,649)	232 (191–2,559)	199 (191–208)
IL-6 after CytoSorb (ng/L)	1,401 (201–14,230)	1,459 (366–8,393)	790 (276–3,462)	173 (165–2,510)	167 (157–176)
*p*-value	<0.001	<0.001	0.01	0.50	0.18
Extraction ratio (%)	26 (18–37)	10 (6–21)	16 (5–21)	9 (2–29)	16 (15–18)

Data are presented as medians and interquartile ranges, and comparisons are made using the Wilcoxon signed-rank test.

**Figure 2:**
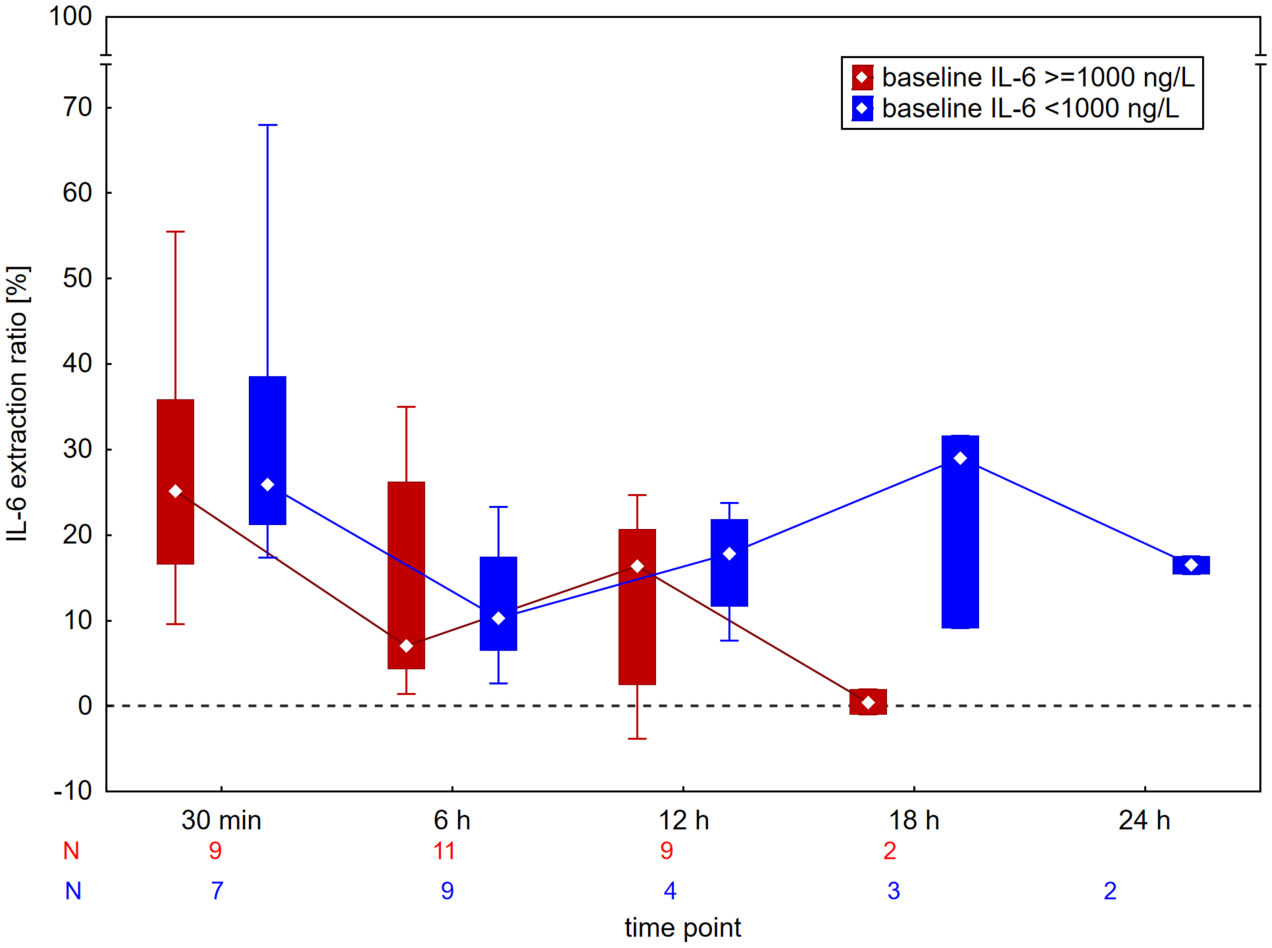
Extraction ratio of interleukin-6 (IL-6) across the CytoSorb adsorber according to baseline IL-6 levels. Data are presented as medians and interquartile ranges. There was no significant difference at any time point.

**Figure 3:**
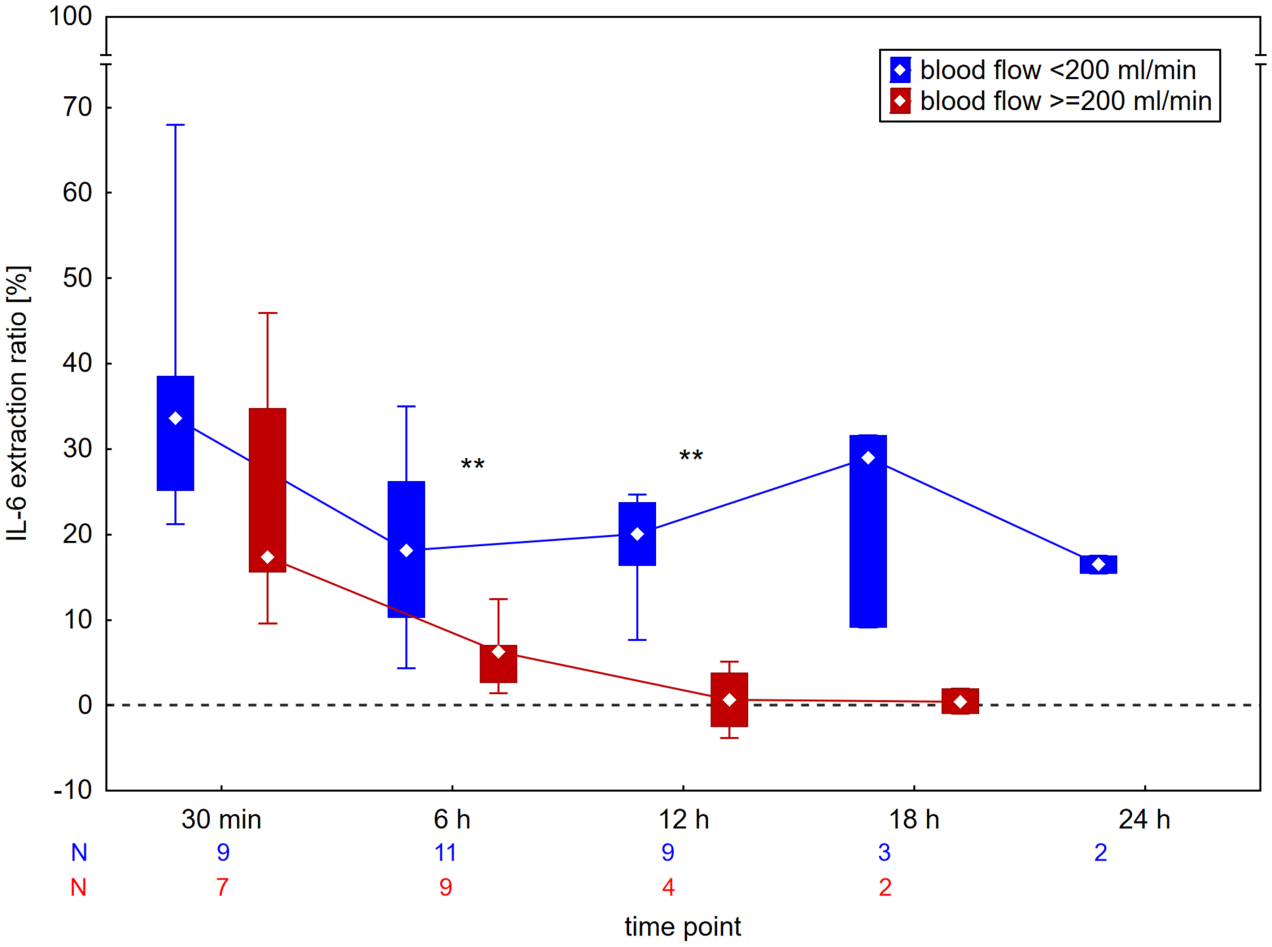
Extraction ratio of interleukin-6 (IL-6) across the CytoSorb adsorber according to blood flow during the procedure. Data are presented as median and interquartile range. All circuits with low blood flow, for which data were available at 18 and 24 h, had low baseline IL-6 levels (<1,000 ng/L). ^**^p < 0.01 for between-group difference.

The median extraction ratios across the CytoSorb adsorber are shown in Table 1. The median extraction ratio was 26% after 30 min and declined to 10% after 6 h. They remained between 9 and 16% up to 24 h; however, data for 18- and 24-h time points were limited and measured at very low IL-6 levels. A comparison of extraction ratios in circuits with high (>1,000 ng/L) and low (<1,000 ng/L) baseline IL-6 is shown in Figure 2; sustained extraction ratios at 18 h were observed only in circuits with low baseline IL-6. A comparison according to blood flow during the procedure is shown in Figure 3. Circuits were divided into high blood flow (≥200 mL/min; 11 circuits, mean blood flow 250 ± 32 mL/min, roughly corresponding to intermittent HD) and low blood flow (<200 mL/min; 10 circuits, mean blood flow 147 ± 18 mL/min, roughly corresponding to continuous HD methods) groups. In the high blood flow group, the extraction ratio was significantly lower after 6 h, when it dropped to a median of 6%, becoming negligible thereafter. In contrast, circuits with low blood flow maintained an extraction ratio of 20% for up to 12 h. The extraction ratios at 18 h and 24 h should be interpreted with caution, as these circuits had low baseline IL-6 levels (<1,000 ng/L). However, the patients had high baseline levels of bilirubin (570 μmol/L) or myoglobin (450,000 μg/L), both of which are known to contribute to saturation of the adsorber.

## Discussion

There are limited data in the literature on the efficiency (extraction ratios) of the CytoSorb, and a call for more data has recently been published (10). Our study is the first clinical report to present CytoSorb extraction ratios over a treatment duration of up to 12 h. We observed sustained adsorption efficiency for up to 12 h only in procedures with low blood flow (<200 mL/min, corresponding to continuous dialysis methods). On the other hand, there was evident adsorber saturation with very low extraction ratios in high blood flow procedures (iHD) already at 6 h. Data beyond 12 h should be interpreted with caution because of the small number of circuits. The overall rapid decrease in IL-6 levels in our patients is probably not primarily because of the adsorption of IL-6, but rather due to intrinsic clearance as the patient’s condition improved. This is illustrated by the case (Circuit 11, Figure 1B) of a patient who was treated with CytoSorb because of hemophagocytic syndrome, developed sepsis during treatment with CytoSorb, and had increasing IL-6 levels over time despite effective removal.

When CytoSorb was first used in brain-dead organ donors, an IL-6 extraction ratio of 28% was reported, which did not change significantly between the first and fourth hours of treatment (5). Later, in a randomized trial by Schaedler et al. (6), the extraction ratio was 18% at 15 min after the start of treatment, declined to below 10% after 1 h, and reached just 5% at 6 h. Recently, in an endotoxemia model, IL-6 plasma clearance significantly decreased over time, decreasing from 25 mL/min (approx. equivalent to 20% extraction ratio at 250 mL/min blood flow) to 10–15 mL/min (approximately 9% extraction ratio) after 5–6 h of treatment with CytoSorb (2).

Our study confirms a good extraction ratio of 26% within the first 30 min, which declined to 10% by 6 h, in agreement with previous studies (2, 5). For the first time, we demonstrate that this lower extraction ratio persists for up to 12 h, or potentially longer, but only in circuits with low blood flow (<200 mL/min). Beyond 12 h, our data are based on a small number of cases with very low or normal baseline IL-6 levels (but very high bilirubin or myoglobin levels) and should be interpreted with caution. The phenomenon of adsorber saturation should depend on the baseline levels of the target substance. However, we did not observe a difference in IL-6 extraction ratios based on baseline IL-6 levels, which is consistent with observations in myoglobin adsorption (11). Conversely, higher blood flows (≥200 mL/min) accelerated adsorber saturation in iHD, with extraction ratios declining significantly to ≤6% by 6 h. These two observations indicate that the adsorption of other substances, which is greater at higher flows, contributes to adsorber saturation, in addition to IL-6, which is consistent with the non-specific adsorption mechanism of CytoSorb. Our results partially support the recently published consensus statement recommending adsorber exchange every 8–12 h for cytokine (8) and myoglobin (9) adsorption. However, this interval appears suitable only for continuous methods, whereas more intensive treatments with higher blood flows most likely require adsorber exchanges every 6–8 h to maintain adsorption efficacy.

Previously published data on myoglobin and bilirubin extraction ratios are similarly consistent. In a small randomized study using continuous methods at 200 mL/min blood flow, the myoglobin extraction ratio was 40% at 30 min but decreased to approximately 10% by 8 h (11). For bilirubin adsorption, obvious saturation with stabilization of serum bilirubin levels was attained within 12 h of CytoSorb treatment in children (12). In adults, one study using continuous methods at 100–150 mL/min blood flow reported a bilirubin extraction ratio of 32% after 10 min, which decreased rapidly to 4.5% after 6 h (13). Another study with similar blood flows showed a 26% extraction ratio at 6 h, with no measurable removal after 12 h (14). In contrast, we previously reported a case of extreme hyperbilirubinemia in which extraction ratios of 15–19% were sustained at 12 and even 24 h of CytoSorb treatment, despite a relatively high blood flow of 200 mL/min (15).

The strengths of our study include its prospective design, systematic blood sampling, and reporting of values directly after the CytoSorb adsorber and not after the dialyzer, as in the majority of other studies. However, limitations exist: the number of circuits included is moderate, especially beyond 12 h, and the dialysis methods and blood flows were variable. Furthermore, some of the patients had very low baseline IL-6 levels, which likely prevented adsorber saturation and artificially increased the extraction ratios. In a few cases, the value after the dialyzer was used instead of the missing value after CytoSorb, which (given the pattern of a slight increase in IL-6 levels over the dialyzer) could slightly decrease the extraction ratio.

In conclusion, our findings showed that IL-6 extraction ratios declined significantly to 6% within 6 h of CytoSorb treatment in treatments with a blood flow of ≥200 mL/min, whereas circuits with a lower blood flow maintained an extraction ratio of approximately 20% for up to 12 h. These results are consistent with the data on bilirubin and myoglobin adsorption. The recent consensus recommendations for an 8–12 h exchange interval for the CytoSorb adsorber (8, 9) should mainly be applied to continuous dialysis methods with low blood flow. In contrast, high-flow modalities, such as iHD, may require more frequent adsorber exchanges to maintain treatment efficacy.

## Data Availability

The raw data supporting the conclusions of this article will be made available by the authors, without undue reservation.
